# Digital Learning in Speech-Language Pathology, Phoniatrics, and Otolaryngology: Interdisciplinary and Exploratory Analysis of Content, Organizing Structures, and Formats

**DOI:** 10.2196/27901

**Published:** 2021-07-27

**Authors:** Yuchen Lin, Christiane Neuschaefer-Rube

**Affiliations:** 1 Clinic for Phoniatrics, Pedaudiology & Communication Disorders University Hospital and Medical Faculty RWTH Aachen University Aachen Germany

**Keywords:** digital learning, e-learning, speech-language pathology, phoniatrics, otolaryngology, communication disorders, mobile phone

## Abstract

**Background:**

The digital revolution is rapidly transforming health care and clinical teaching and learning. Relative to other medical fields, the interdisciplinary fields of speech-language pathology (SLP), phoniatrics, and otolaryngology have been slower to take up digital tools for therapeutic, teaching, and learning purposes—a process that was recently expedited by the COVID-19 pandemic. Although many current teaching and learning tools have restricted or institution-only access, there are many openly accessible tools that have gone largely unexplored. To find, use, and evaluate such resources, it is important to be familiar with the structures, concepts, and formats of existing digital tools.

**Objective:**

This descriptive study aims to investigate digital learning tools and resources in SLP, phoniatrics, and otolaryngology. Differences in content, learning goals, and digital formats between academic-level learners and clinical-professional learners are explored.

**Methods:**

A systematic search of generic and academic search engines (eg, Google and PubMed); the App Store; Google Play Store; and websites of established SLP, phoniatrics, and otolaryngology organizations was conducted. By using specific search terms and detailed inclusion and exclusion criteria, relevant digital resources were identified. These were organized and analyzed according to learner groups, content matter, learning goals and architectures, and digital formats.

**Results:**

Within- and between-learner group differences among 125 identified tools were investigated. In terms of content, the largest proportion of tools for academic-level learners pertained to anatomy and physiology (60/214, 28%), and that for clinical-professional learners pertained to diagnostic evaluation (47/185, 25.4%). Between groups, the largest differences were observed for anatomy and physiology (academic-level learners: 60/86, 70%; clinical-professional learners: 26/86, 30%) and professional issues (8/28, 29% vs 20/28, 71%). With regard to learning goals, most tools for academic-level learners targeted the performance of procedural skills (50/98, 51%), and those for clinical-professional learners targeted receptive information acquisition (44/62, 71%). Academic-level learners had more tools for supporting higher-level learning goals than clinical-professional learners, specifically tools for performing procedural skills (50/66, 76% vs 16/66, 24%) and strategic skills (8/10, 80% vs 2/10, 20%). Visual formats (eg, pictures or diagrams) were dominant across both learner groups. The greatest between-group differences were observed for interactive formats (45/66, 68% vs 21/66, 32%).

**Conclusions:**

This investigation provides initial insights into openly accessible tools across SLP, phoniatrics, and otolaryngology and their organizing structures. Digital tools in these fields addressed diverse content, although the tools for academic-level learners were greater in number, targeted higher-level learning goals, and had more interactive formats than those for clinical-professional learners. The crucial next steps include investigating the actual use of such tools in practice and students’ and professionals’ attitudes to better improve upon such tools and incorporate them into current and future learning milieus.

## Introduction

### Background

The digital age has introduced tremendous changes and emerging opportunities in teaching and learning, especially in the health care environment. Buzzwords such as *eHealth*, *digital health*, mobile health (*mHealth*), *e-learning*, *digital learning*, and *m-learning* are increasingly enriching the medical language and have infused clinical teaching and practice with new vocabulary and concepts. The terms *eHealth* or *digital health* have often been used to refer to a broad spectrum of information communication technology applications in which information can be processed or exchanged electronically and can be used to support patient treatment and care; *mHealth* refers to these processes and apps on mobile devices such as tablets, smartphones, or smartwatches [1,2]. On the other hand, *e-learning* or *digital learning* are broad terms that can be used to describe a wide range of methods in which digital media, internet, and information and communication technologies are used for teaching and learning purposes to optimize knowledge creation and reproduction, interpersonal exchange, or collaborative work; the term *m-learning* thus refers to the implementation of these processes on mobile devices [3-6]. However, because of rapid changes in technology and didactic approaches, definitions often become obsolete faster than they can be created [7]. The emergence and continuous renewal of such concepts and digital possibilities not only demonstrate the enormous scope and potential for development of digital solutions but also highlight how the knowledge and skills required by current and future clinicians are gradually expanding to include technical skills.

Given the (1) increasing number of portable devices and technologies, (2) increasing accessibility to information, and (3) new generations of learners who process information in a manner that is different from prior generations, “...the issue is not whether we adopt these new technologies but whether we make the most of the opportunities they provide” [8]. Moreover, given the growing aging population and well-reported shortage of health care workers worldwide, digital solutions offer potential avenues for increasing equitable health care accessibility [9-13]. Digital skills will likely become a prerequisite for future health professionals, who will play a major role in educating patients on digital health literacy and optimizing digital patient-centered care [14,15]. It is recognized worldwide that current and future health professionals must be equipped for learning and medical practice in an increasingly digitalized health care system [16,17]. In essence, “[w]e have to prepare students for jobs that have not yet been created, technologies that have not yet been invented and problems that we don’t yet know will arise” [18]. Such a sentiment is especially relevant in light of the COVID-19 pandemic, which has pushed the discussion of digital learning and digital health care solutions and alternatives to the forefront [19-23].

In the interdisciplinary fields of speech-language pathology (SLP), phoniatrics, and otolaryngology, digital possibilities offer great potential. Professionals in these fields collaboratively treat disorders and disabilities affecting speech, language, voice, hearing, and the ability to communicate. The importance and benefit of interdisciplinary education within these fields cannot be understated; in fact, interdisciplinary education will play a significant role in *future-proofing* health professional curricula moving forward [24-27]. Moreover, digital tools can contribute to enhancing such collaborative opportunities and are already beginning to engage other, traditionally more technical fields (eg, informatics and engineering) [25,28]. Given the World Health Organization’s estimate of over one billion people worldwide living with a disability that often affects their functional communication, it is crucial that current and future professionals in these fields are well prepared to advance their knowledge, skills, and coordinated patient care through new digital solutions [29]. Thus, it can be useful to investigate current digital resources collectively across these fields.

Literature has shown that research and outcomes for digital solutions in these fields are only just beginning to emerge relative to other medical fields [30,31]. That is not to say, however, that tools and applications do not already exist. Augmentative and alternative communication devices (eg, speech-generating tablets) and mathematical-linguistic language modeling are just some examples of digital support technologies that are already well established in the field [32,33]. There is also an increasing number of emerging digital applications to assist with diagnostic evaluations and therapeutic exercises; however, knowledge of these tools and their quality appears to be uncertain [34,35]. Given that students and professionals who treat communication disorders have overall reported positive attitudes toward eHealth and a desire for more digital learning opportunities, it is crucial that digital tools are more critically assessed and deliberately integrated into clinical education and professional development [35-37]. To begin this process, it can be helpful to first investigate existing digital e-learning tools. Although it currently appears that many digital learning tools are institution-specific or have restricted access, there is a notable plethora of digital learning resources relevant to the abovementioned interdisciplinary fields with easier accessibility or freely available. These have largely been unexplored in the literature and have yet to be assessed for quality. However, the current range of digital tools is broad and heterogeneous, making it difficult to fully comprehend their purpose or use [37,38].

### Objectives

To effectively find, use, evaluate, and incorporate such resources and tools into learning and teaching scenarios, it is important to be familiar with the structures, concepts, and formats of existing digital learning resources. This study seeks to (1) investigate the current scope of digital tools and resources with free or good accessibility across the interdisciplinary fields of SLP, phoniatrics, and otolaryngology and (2) specifically explore potential differences between resources available for academic-level learners versus clinical-professional learners in terms of content, learning goal, or format. Importantly, this initial study does not aim to investigate the quality of the tools, although this is a necessary next step. It is worth mentioning that given the fast-paced nature of technological development, the number and scope of digital tools and resources at any given time are changing. This investigation was based on a search conducted and updated in the autumn of 2020.

## Methods

### Electronic Search and Inclusion and Exclusion Criteria

A systematic search of Google; Google Scholar; EbscoHost (including PubMed and Medline); Livivo; the App Store, Google Play Store; and established SLP, phoniatrics, and otolaryngology foundation websites was conducted. The foundations and regulating bodies whose websites were searched included the American Speech-Language-Hearing Association, Union of the European Phoniatricians, the International Federation of Oto-Rhino-Laryngological Societies, the European Federation of Oto-Rhino-Laryngology Societies, and the American Academy of Otolaryngology-Head and Neck Surgery. The keywords used were *e-learning* OR *digital learning* AND either *speech pathology*, *speech-language pathology*, *phoniatrics*, *ENT medicine*, and *otolaryngology*.

Inclusion criteria included were as follows:

- The presented information should be relevant for students and professionals in the interdisciplinary fields of SLP, phoniatrics, and otolaryngology.
- The resource should either be openly accessible or have good accessibility (eg, could require account creation but no institution-specific restricted access).
- There is evidence of clinician or physician involvement in resource development.
- The resource should be in the English or German language.

Exclusion criteria included tools or resources used solely for clinical purposes (eg, therapy apps) or specifically for patient use and blogs. Although there is evidence that professional blogs serve as a significant source of information and exchange for practicing professionals and even students, it was not within the scope of this study to identify the full range of professional blogs [39].

### Organizing Structures

#### Overview

To construct a more comprehensible organization for a broad range of available resources, digital tools were specifically analyzed according to (1) learner groups, (2) content areas, (3) learning goals and architectures, and (4) formats.

These organizing structures have commonly been referred to in multimedia learning theories and their applications in other fields [40-43]. Each of these organizing structures is defined in more detail.

#### Learner Groups

In health professional education, there are several ways to differentiate among learner groups. These include, among others, distinctions between preclinical and clinical learners, trainees and attendees, academic introductory and advanced learners, or student clinicians and working professionals [44-46]. These distinctions can vary depending on the specific institution, context, profession, or educational system in a country. With these differences in mind, for the purposes of this study, we have broadly differentiated between the following learner groups, as described below.

#### Academic-Level Learners

This includes those who have introductory and advanced theoretical knowledge with initial clinical experience. Digital resources and tools were allocated to the academic-level learner group when content consisted of introductory information (eg, basic introductions to anatomy and physiology, pathologies, or treatment approaches) or when the content of the resource was explicitly referred to as appropriate for academic-level learners.

#### Clinical-Professional Learners

This includes residents, clinical fellows, and working professionals whose focus is on the clinical integration of knowledge and skills. Residents, clinical fellows, and working professionals were also deliberately grouped together because they shared many overlapping digital resources. Resources and tools that addressed the advanced integration of diagnostic or treatment strategies or that explicitly identified the content as appropriate for clinical fellows, residents, or professionals were allocated to the clinical-professional learner group.

This study also aims to investigate whether there were differences in digital tools and resources available between these two broad learner groups in terms of content, learning goals, and formats.

#### Content Areas

For the following investigation, digital tools, and resources were grouped into the following broad categories, as these were the observed prominent reoccurring content areas, which are also common to all the interdisciplinary fields involved with communication disorders: anatomy and physiology, diagnostic evaluation, pathology, treatment, professional issues, and other (eg, networking).

#### Learning Goals and Architectures

According to the cognitive theory of multimedia learning by Mayer [47], e-learning goals can be primarily divided into *inform* versus *perform* goals. *Informing* goals focus on the transmission of information and may not specify any expectations for the acquisition of new skills, whereas goals focused on *performing* do specify new skills to be attained and can be further divided into performing *procedural tasks* and *strategic tasks*. Procedural tasks encourage response strengthening and thus promote near transfer, whereas strategic tasks encourage knowledge instruction, which promotes the far transfer and, ideally, the application of knowledge to other contexts [40]. These learning goals are closely aligned with e-learning architectures, which include *receptive*, *directive*, and *guided discovery*. These architectures provide a broad framework for understanding the nature and purpose of learning interactions. Specifically, *inform* learning goals are *receptive* (low behavioral engagement), the learning goal of *performing procedural skills* is *directive* (medium behavioral engagement), and the learning goal of *performing strategic skills* promotes *guided discovery* (high behavioral engagement).

#### Formats

Content formats of digital learning resources and tools refer to the specific configuration by which information is displayed. Content formats can vary according to sensory modality, level of interaction, level of virtuality, level of mediality, and flexibility of synchronous or asynchronous use [48]. These dimensions are not always clearly defined, as they can also be affected by the specific way in which a digital tool or resource is implemented or used for learning purposes (eg, a simulation could be used synchronously or asynchronously or may have varying levels of interactivity depending on the specific exercise performed or the learning goal targeted). For the purposes of this study, formats have been organized into (1) verbal, (2) visual, and (3) interactive presentation forms, as suggested by Arnold et al [41]. Verbal formats include audio- and text-based information or activities such as websites, e-books, or podcasts. Examples of visual formats include static pictures or diagrams, videos, 3D models or manipulatives, portals, or apps that integrate multiple verbal or visual formats. Examples of interactive formats include simulations, social networking channels, web-based courses, serious games, 3D worlds, or dynamic apps that include interactive elements. Notably, the distinctions among these categories are somewhat fluid and overlapping (eg, a website could have visual and verbal elements and even contain interactive case scenarios). Although it is not within the scope of this investigation to review all existing digital formats, relevant formats for the digital tools and resources identified in this study are discussed in greater depth in the *Results* and *Discussion* section.

Systematic searches and subsequent analyses were performed by 2 authors, a certified speech-language pathologist (YL) and qualified phoniatrician and otorhinolaryngologist (CNR), both of whom have experience in clinical practice, teaching, and research. It is important to note that internet search results can change depending on a user’s browser type, cookie settings, search history, exact location, time, and more [49]. Thus, searches were conducted in the incognito mode on two institution-owned computers. Tools with relevant references underwent two additional iterative searches. The authors independently screened and analyzed the tools, and any disagreements in the analysis among categories were resolved through discussion.

## Results

### Overview

**Figure 1:**
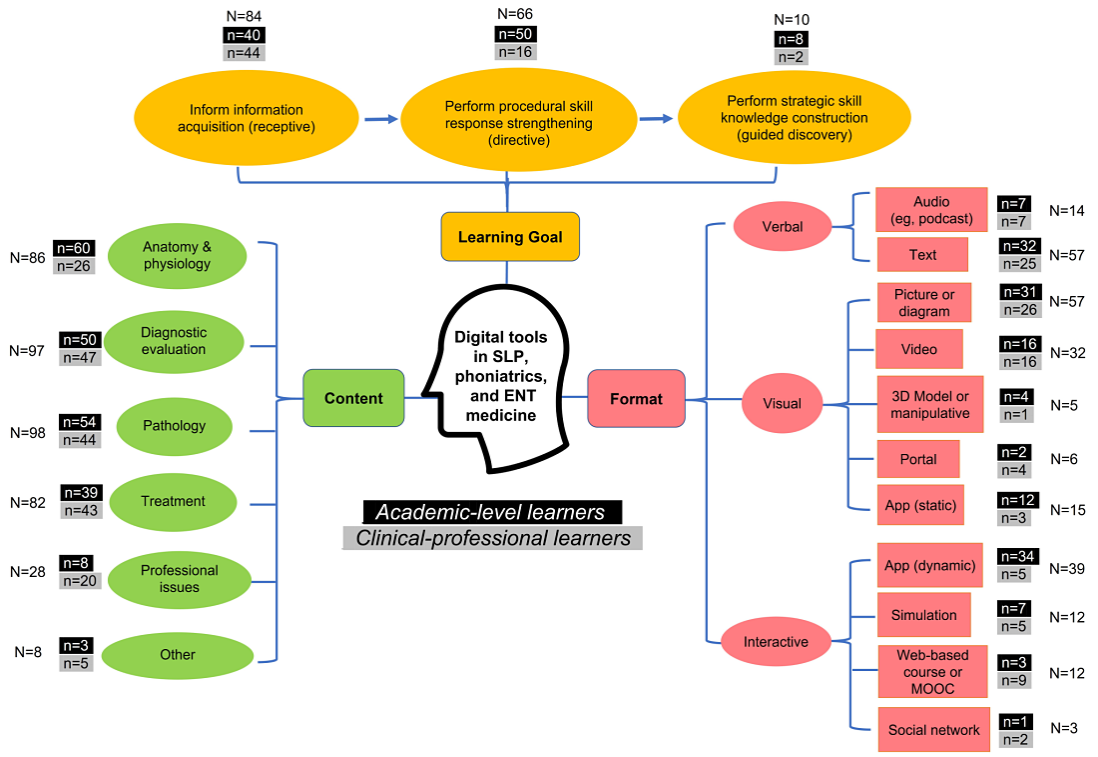
Summary of digital tools and resources organized according to the learner group, content, learning goal, and format. ENT: ear, nose, throat; MOOC: massive online open course; SLP: speech-language pathology or pathologist.

A total of 125 digital tools and resources that met all the inclusion and exclusion criteria were identified. These are listed in Multimedia Appendix 1. Of these tools, 78.4% (98/125) were appropriate for academic-level learners (introductory and advanced theoretical knowledge with minimal clinical experience) and 49.6% (62/125) were appropriate for clinical-professional learners (eg, residents, clinical fellows, and working professionals), with a 28.8% (35/125) overlap between the two groups. Upon categorizing each of the three components analyzed (ie, content, learning goal, and format), there were often tools with overlapping categories (eg, a digital resource could contain multiple content areas or multiple formats). These overlaps were included in the frequency counts during data analysis to reflect the appropriate proportion of tools specifically fulfilling the indicated category. The full distribution of tools denoted by frequencies (eg, number of tools) and organized according to content, learning goal, and formats is shown in Figure 1.

### Content

#### Overview

Content was broadly divided into the topics of anatomy and physiology, diagnostic evaluation, pathology, treatment, professional issues, and other (eg, networking). Across the 399 total frequency counts for content areas including overlaps, 24.6% (98/399) pertained to pathology, 24.3% (97/399) to diagnostic evaluation, 21.6% (86/399) to anatomy and physiology, and 20.5% (82/399) to treatment. Professional issues and other subjects comprised 7% (28/399) and 2% (8/399) of the total resources, respectively.

#### Within-Group Differences

The distribution of tools within each learner group is represented as a frequency count, followed by percentages of the total number of tools and resources for that specific learner group. Most tools for academic-level learners consisted of content pertaining to anatomy and physiology (60/214, 28%), pathology (54/214, 25.3%), diagnostic evaluation (50/214, 23.4%), and treatment (39/214, 18.2%). Tools pertaining to professional issues and other subjects (eg, networking) were far fewer in number. Tools and resources for the clinical-professional learner group mostly fell within the content categories of diagnostic evaluation (47/185, 25.4%), pathology (44/185, 23.7%), and treatment (43/185, 23.3%). These data and further details are summarized in Table 1.

**Table 1:** Distribution of digital tools within each learner group according to content.

Content category	Academic-level learners (n=214), n (%)	Clinical-professional learners (n=185), n (%)
Anatomy and physiology	60 (28)	26 (14.1)
Diagnostic evaluation	50 (23.4)	47 (25.4)
Pathology	54 (25.3)	44 (23.7)
Treatment	39 (18.2)	43 (23.3)
Professional issues	8 (3.7)	20 (10.8)
Other	3 (1.4)	5 (2.7)

#### Between-Group Differences

**Figure 2:**
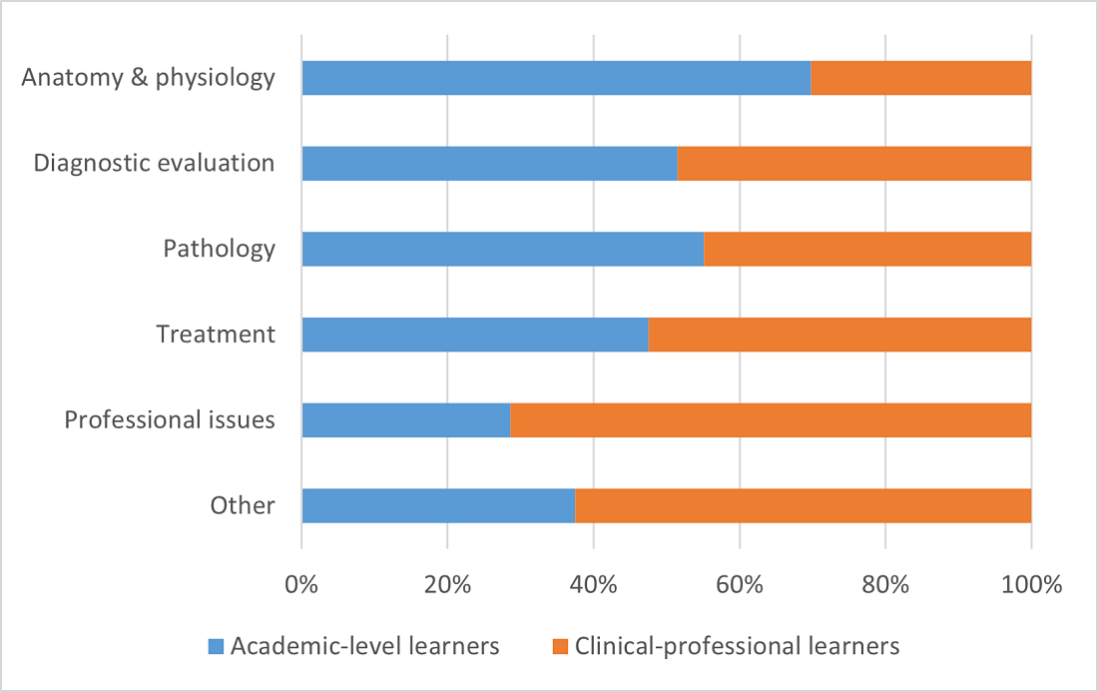
Digital tools available between learner groups according to content.

The distribution of tools between academic-level learners and clinical-professional learners is presented as frequency counts and percentages of the total number of tools and resources for a specific content category. Data are always presented as academic-level learners versus clinical-professional learners. Some of the largest differences in terms of digital tools and resources between academic-level learners and clinical-professional learners were observed for the content areas of (1) anatomy and physiology, where academic-level learners had a greater proportion of resources (60/86, 70% vs 26/86, 30%) and (2) professional issues (8/28, 29% vs 20/28, 71%) and (3) other resources such as networking sites (3/8, 37% vs 5/8, 63%). There was a relatively similar number of tools for diagnostic evaluation between the 2 learner groups (50/97, 51% vs 47/97, 49%), slightly more tools relating to pathology for academic-level learners (54/98, 55% vs 44/98, 45%), and slightly fewer tools for them that related to treatment (39/82, 48% vs 43/82, 52%). These data are graphically summarized in Figure 2.

### Learning Goal

#### Overview

Learning goals were differentiated among those with a (1) *inform* through information acquisition focus and *receptive* learning architecture, (2) to *perform* procedural skills focus and *directive* architecture, and (3) those with a *perform* strategic skills focus and *guided discovery* architecture. Furthermore, 52.5% (84/160) of tools had the learning goal of *receptive* information acquisition; 41.2% (66/160) had the learning goal of performance of procedural skills, a more *directive* learning architecture. Only 6.3% (10/160) of tools supported the highest-level learning goal of performance of strategic skill, which would encourage *guided discovery*.

#### Within-Group Differences

Approximately half of the digital tools and resources for academic-level learners (50/98, 51%) had the learning goal of performing a procedural skill and thus had a more *directive* learning architecture. A large proportion of the digital resources and tools for the academic-level–learner group (40/98, 41%) also had the learning goal of receptive information acquisition, and only a few targeted the learning goal of performing a strategic skill through the learning architecture of *guided discovery*.

Most tools and resources for clinical-professional learners served the purpose of information acquisition through *receptive* learning architectures (44/62, 71%). A large proportion of tools (16/62, 26%) aimed to perform procedural skills through a *directive* architecture, and very few tools aimed to perform strategic skills through the process of *guided discovery*. These data and details are summarized in Table 2.

**Table 2:** Distribution of digital tools within each learner group according to learning goals.

Learning goal	Academic-level learners (n=98), n (%)	Clinical-professional learners (n=62), n (%)
Inform (information acquisition), receptive	50 (41)	44 (71)
Perform (procedural skill), directive	40 (51)	16 (26)
Perform (strategic skills), guided discovery	8 (8)	2 (3)

#### Between-Group Differences

**Figure 3:**
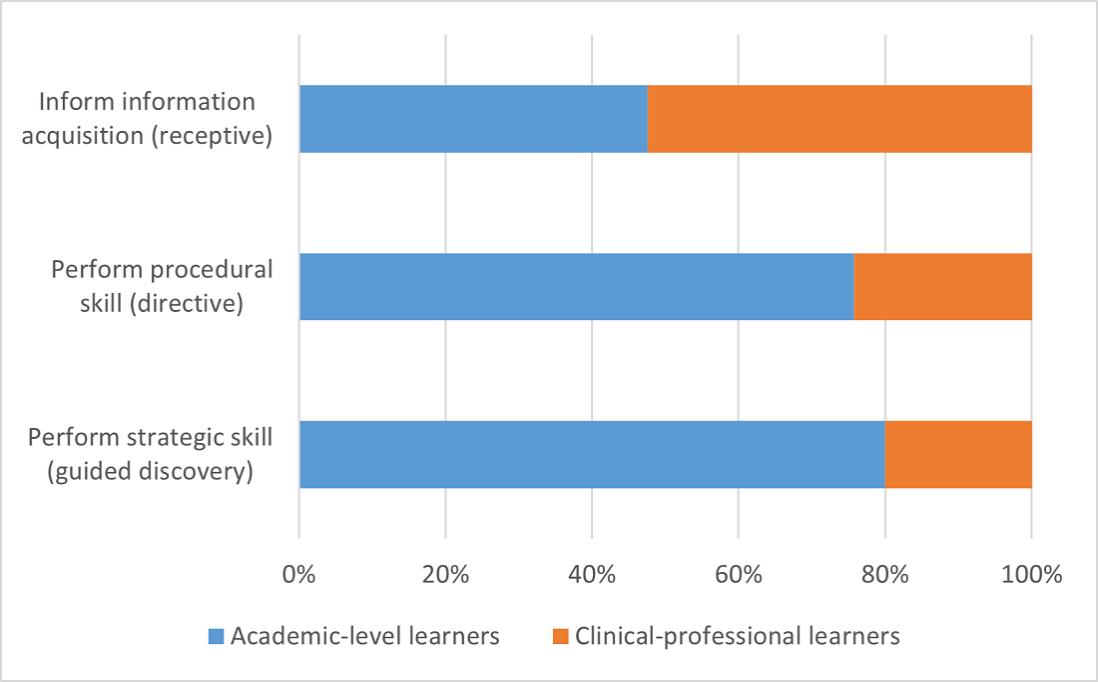
Digital tools available between learner groups according to learning goals.

The distribution of tools between academic-level learners and clinical-professional learners is presented as frequency counts and percentages of the total number of tools and resources for a specific learning goal type. Data are always presented as academic-level learners versus clinical-professional learners. It appears that as the learning goal becomes more advanced, that is from informing through *receptive* information acquisition to performing a strategic skill for *guided discovery*, we observed greater differences in the proportions of tools between academic-level learners and clinical-professional learners. Although it appears that there is a relatively close number of digital tools and resources for both learner groups that support the informing learning goal (40/84, 48% vs 44/84, 52%), academic-level learners have a much greater proportion of the tools that support performing a procedural skill (50/66, 76% vs 16/66, 24%) and those that support performing a strategic skill (8/10, 80% vs 2/10, 20%) than their clinical-professional counterparts. These data are graphically summarized in Figure 3.

### Format

#### Overview

**Figure 4:**
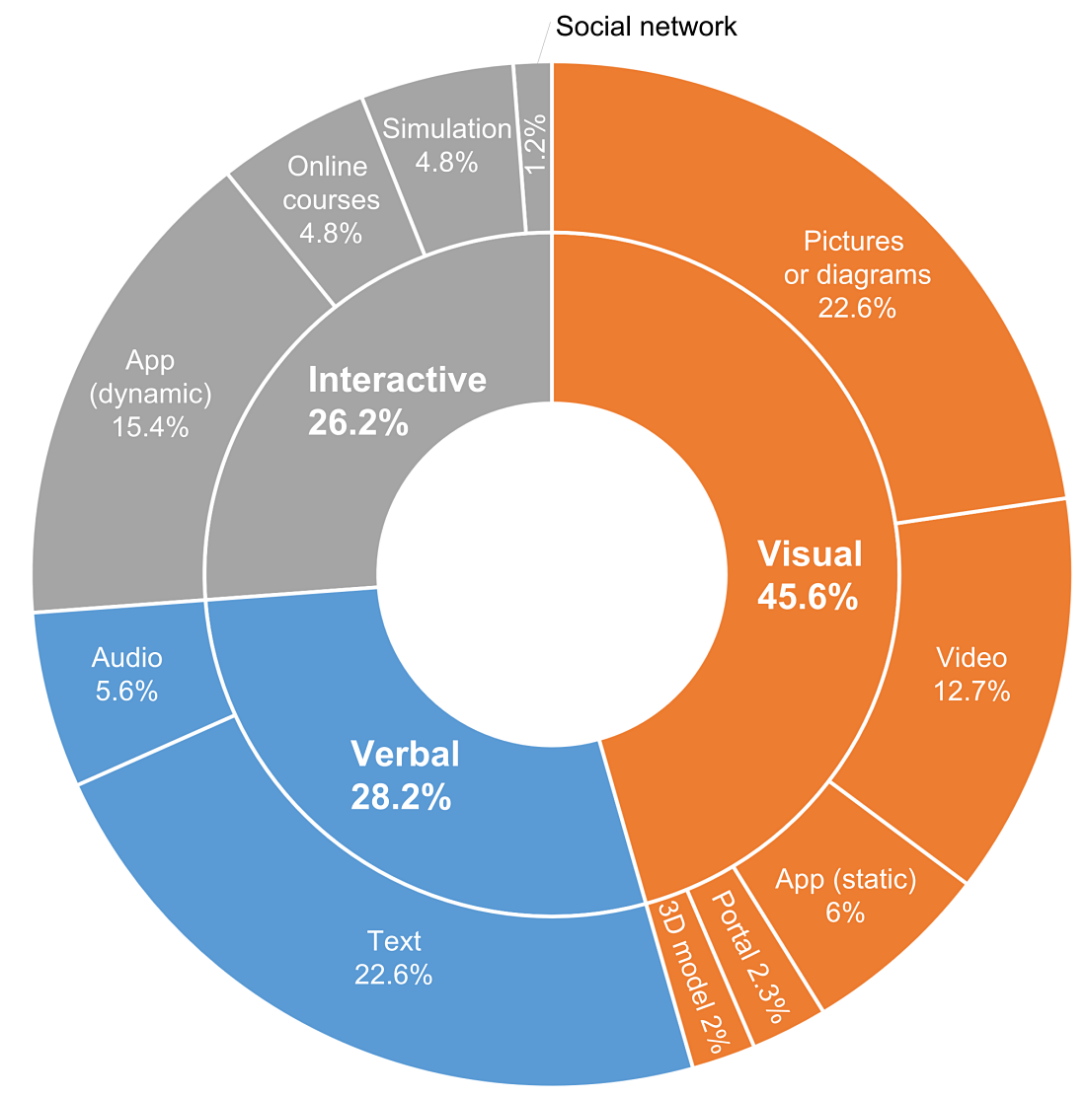
Summary of the distribution of digital tools according to format types.

Digital tools and resources were broadly divided into verbal, visual, and interactive formats. These were further subdivided on the basis of specific format types (eg, video, 3D model, and simulation). Only the formats that were present in the range of the investigated tools and resources were included in the study. There are certainly numerous other existing formats (eg, serious games, and 3D worlds) that were not represented in the sample as they—to the best of our knowledge—do not yet exist or are not yet readily available for the fields of SLP, phoniatrics, or otolaryngology. Overall, a large majority of digital tools were visual in nature (115/252, 45.6%), followed by verbal (71/252, 28.2%), and interactive (66/252, 26.2%). When each of these components was separated further, it was observed that large and equal proportions of the digital tools consisted of pictures or diagrams (57/252, 22.6%) and text (57/252, 22.6%). There were also a notable portion of dynamic apps (39/252, 15.4%) and videos (32/252, 12.7%). The distribution of the different formats and further details are shown in Figure 4.

#### Within-Group Differences

Visual formats comprised the largest proportion of formats overall for academic-level learners, with a large proportion of pictures or diagrams (31/149, 20.8%), followed by videos (16/149, 10.7%). The next largest subgroup of formats consisted of interactive formats. Notably, this subgroup predominantly consisted of dynamic apps. Simulations, web-based courses or massive online open courses (MOOCs), and social networks only comprised 7.4% (11/149) of the total frequency count altogether. Here, it is useful to briefly mention that apps were deliberately separated into *static* and *dynamic* apps. Static apps were defined as apps that involved minimal interaction (eg, simple text and visuals in an app form with little to no animation or clickable interactive elements), whereas dynamic apps involved a higher level of virtuality and interaction (eg, animations, virtuality, and more integrated multimedia). In terms of verbal formats for academic-level learners, the majority of the digital tools were text-based (32/149, 21.5%).

For clinical-professional learners, visual formats comprised the largest proportion of the digital resources and tools collected. A quarter of the total number of tools consisted of pictures or diagrams (26/103, 25.2%), followed by a notable proportion of videos (16/103, 15.5%). Other visual formats comprised 7.8% (8/103) of all the tools together. Verbal formats comprised the second largest group of formats, with most being text-based (25/103, 24.3%) and few consisting of audio formats. Finally, interactive formats comprised the smallest proportion of tools and resources for clinical-professional learners. Web-based courses or MOOCs (often used for continuing education credits) accounted for 8.7% (9/103) of tools, followed by equal proportions of dynamic apps and simulations (both 5/103, 4.9%). The data and further details are summarized in Table 3.

**Table 3:** Distribution of digital tools within each learner group according to formats.

Format			Academic-level learners (n=149), n (%)		Clinical-professional learners (n=103), n (%)
**Verbal**					
	Audio (eg, podcast)	7 (4.7)		7 (6.8)	
	Text	32 (21.5)		25 (24.3)	
**Visual**					
	Pictures or diagrams	31 (20.8)		26 (25.2)	
	Video	16 (10.7)		16 (15.5)	
	3D model or manipulative	4 (2.7)		1 (1)	
	Portal	2 (1.3)		4 (3.9)	
	App (static)	12 (8.1)		3 (2.9)	
**Interactive**					
	App (dynamic)	34 (22.8)		5 (4.9)	
	Simulation	7 (4.7)		5 (4.9)	
	Web-based course or MOOC^a^	3 (2)		9 (8.7)	
	Social network	1 (0.7)		2 (1.9)	

^a^MOOC: massive online open course.

#### Between-Group Differences

**Figure 5:**
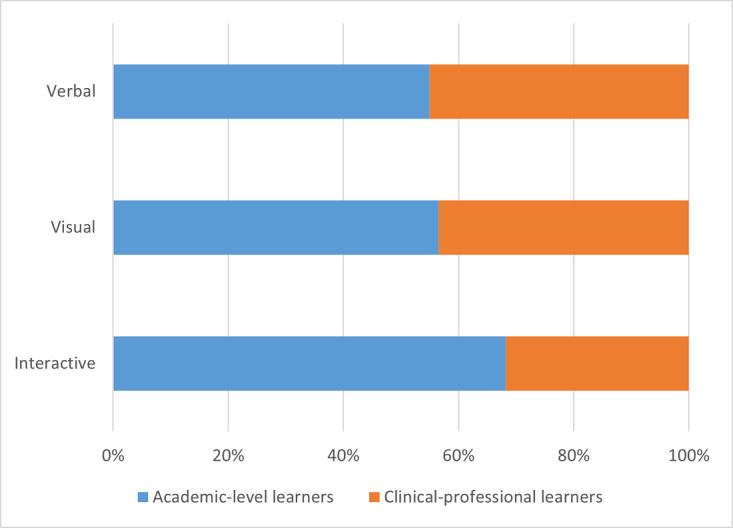
Digital tools available between learner groups according to format types.

**Figure 6:**
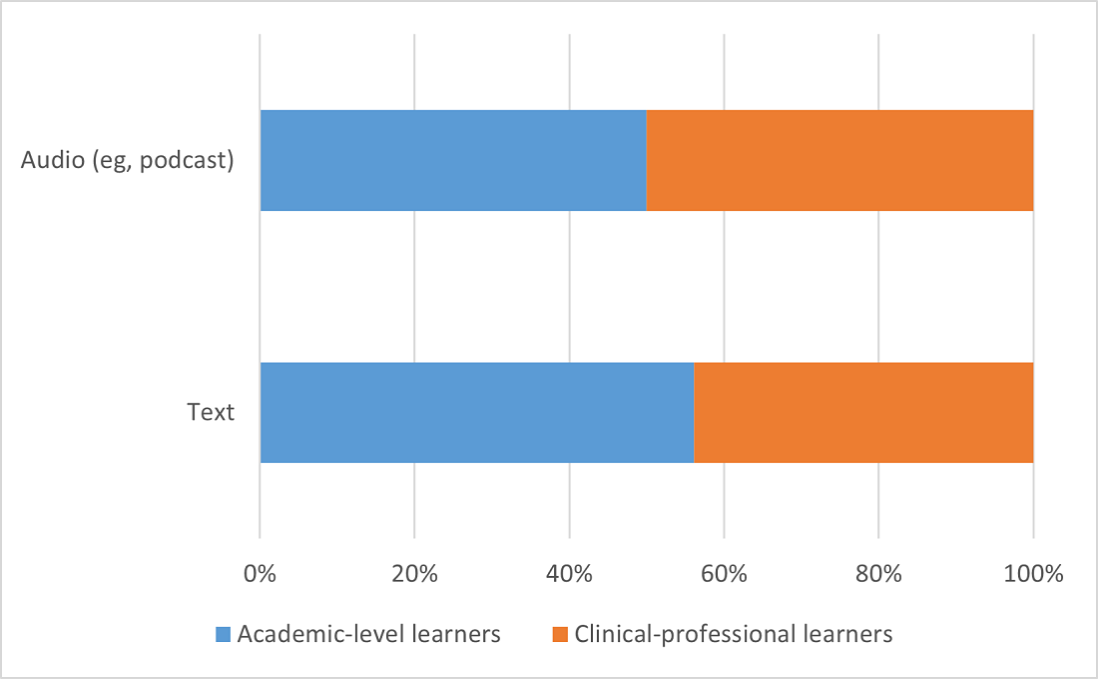
Digital tools available between learner groups in verbal formats.

**Figure 7:**
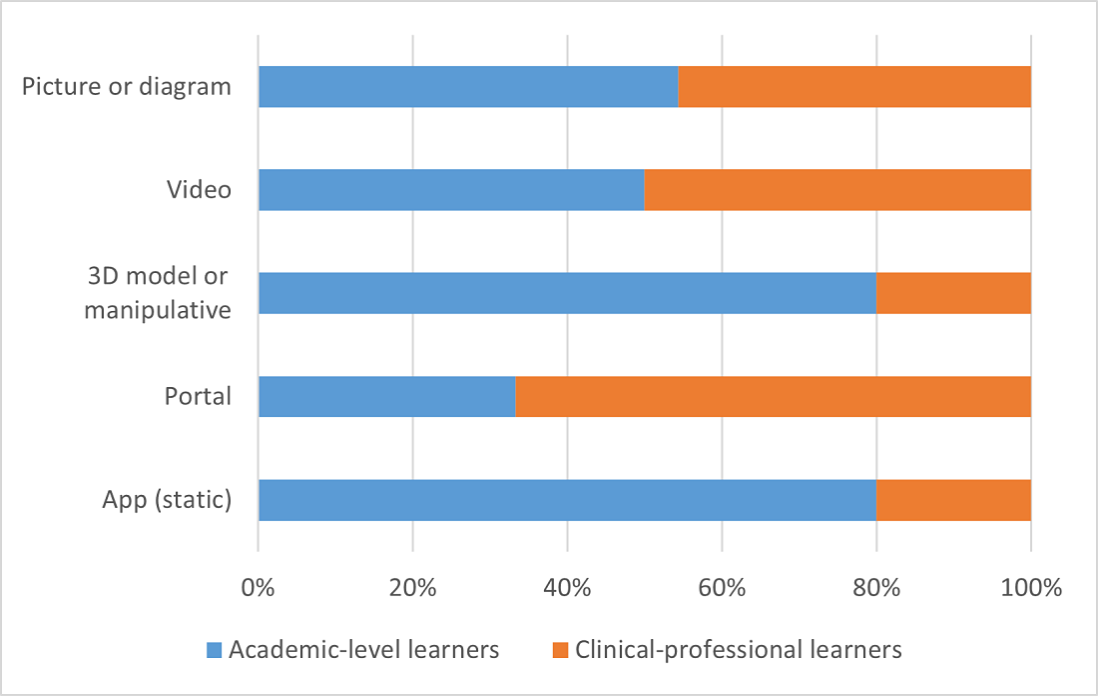
Digital tools available between learner groups in visual formats.

**Figure 8:**
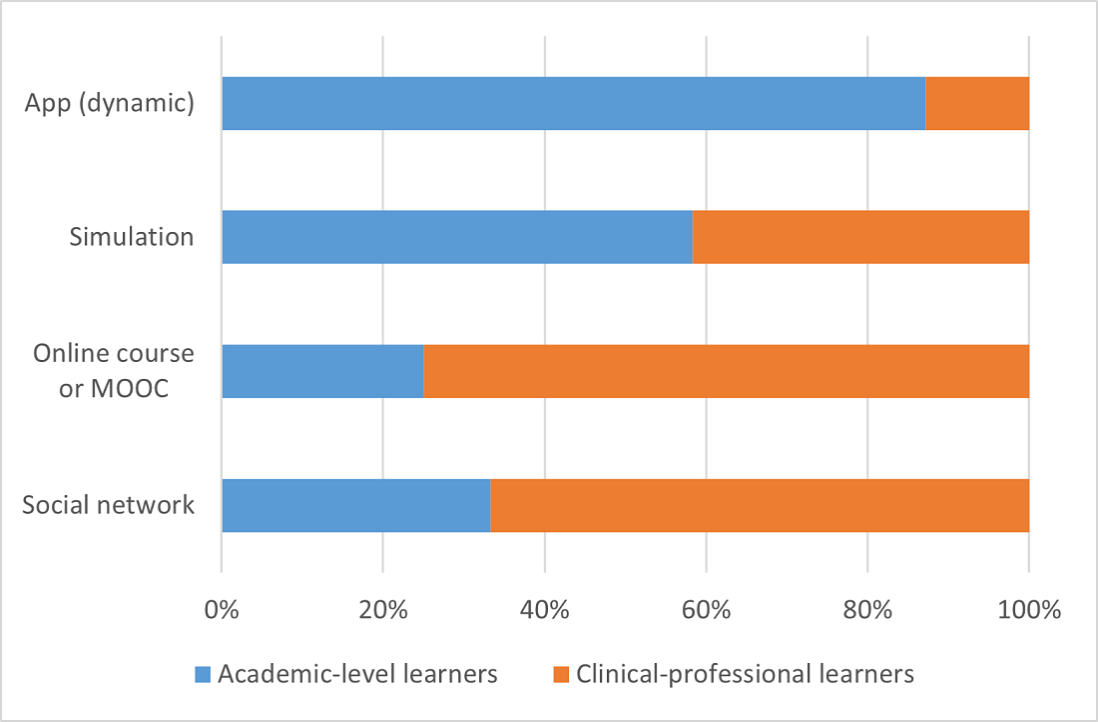
Digital tools available between learner groups in interactive formats. MOOC: massive online open course.

The distribution of tools between academic-level learners and clinical-professional learners is represented as frequency counts and percentages of the total number of tools and resources for a specific format type. Data are always presented as academic-level learners first versus clinical-professional learners second. Among the subordinate categories of verbal, visual, and interactive formats, academic-level learners had only slightly more verbal (39/71, 55% vs 32/71, 45%) and visual formats (65/115, 57% vs 50/115, 43%) than clinical-professional learners, although this proportional difference was much more pronounced with interactive formats (45/66, 68% vs 21/66, 32%). Within the subcategory of verbal formats, there was an equal proportion of audio formats across both academic-level and clinical-professional learners (7/14, 50% vs 7/14, 50%) and slightly more text formats for academic-level learners than for clinical-professional learners (32/57, 56% vs 25/57, 44%). Within the subcategory of visual formats, the greatest differences between the 2 learner groups were noted for 3D models (4/5, 80% vs 1/5, 20%) or manipulatives and for static apps (12/15, 80% vs 3/15, 20%). Academic-level learners had fewer tools and resources in a portal (2/6, 33% vs 4/6, 67%), slightly more tools in picture or diagram formats (31/57, 54% vs 26/57, 46%), and the same proportion of video formats (16/32, 50% vs 16/32, 50%) than their clinical-professional learner counterparts. Within the subcategory of interactive formats, the greatest difference was observed in the proportion of dynamic app formats (34/39, 87% vs 5/39, 13%), although a notable difference was also seen in web-based courses or MOOCs (3/12, 25% vs 9/12, 75%) and social networks (1/3, 33% vs 2/3, 67%), for which there were more resources for the clinical-professional learner group. Finally, academic-level learners had a slightly greater proportion of digital tools with simulation formats than their clinical-professional learner counterparts (7/12, 58% vs 5/12, 42%). These data are graphically summarized in Figure 5. Figure 6, Figure 7, and Figure 8 depict the verbal, visual, and interactive tools between the 2 learner groups, respectively.

## Discussion

### Principal Findings

To the best of our knowledge, this is the first study to investigate openly accessible tools within the interdisciplinary context of SLP, phoniatrics, and otolaryngology. Although it appears that significant literature is focused on the implementation of e-learning or digital learning solutions at specific institutions, it is also crucial to analyze tools with greater public accessibility, as despite their growing number and range, their quality remains unassessed and are nevertheless sources of learning and teaching that are also being used.

This initial investigation of tools revealed that overall, there appears to be a greater number of tools and resources for academic-level learners than for clinical-professional learners, although there was also a considerable amount of overlap between them (n=35). These tools contained wide-ranging subject matter, targeted different learning goals, and were presented in various digital formats. Below, the implications of the results for each of these aspects are explored in greater depth.

### Content

Overall, between the 2 learner groups, content categories appeared to primarily focus on the topics of pathology, diagnostic evaluation, anatomy and physiology as well as treatment. As these primary subjects comprise the bulk of necessary clinical knowledge, for which there are frequently new findings and developing research, this is not particularly surprising. The smallest proportion of digital tools and resources were dedicated to *other* content, namely those focused on field-specific networking sites or exchange sites. Given the predominance of large networking channels such as Facebook, Twitter, Instagram, and professional blogs, it could be that these *other* resources simply are not as commonly used. Interestingly, when looking within each learner group, the subject with the greatest percentage of tools for academic-level learners was focused on anatomy and physiology. This makes sense as these learners are still developing foundational conceptual knowledge to understand how pathologies affect these anatomical structures and their normal functioning. On the other hand, for the clinical-professional learner group, the largest percentage of tools was focused on diagnostic evaluation. Given that there is constantly new research emerging regarding new diagnostic measures, pathologies, and their treatment strategies, these results are not surprising. Between the two learner groups, we observed that the number of tools focused on anatomy and physiology is notably smaller for the clinical-professional learner group, whereas the number of tools for professional issues is greater. This makes sense given that clinical-professional learners should already be familiar with such foundational concepts of anatomy and physiology and must navigate professional issues such as interdisciplinary exchange or work effectiveness on a day-to-day basis. However, the dearth of tools and resources for academic-level learners regarding professional issues may highlight an area that needs to be bolstered in communication sciences and disorders education; in fact, studies have shown that students often arrive at their clinical placements unprepared for the combination of clinical and professional responsibilities that comprise their day-to-day work [50-53]. Therefore, a greater incorporation of digital tools and resources or curricular content addressing these professional issues for the academic-level learners would be beneficial in the future.

### Learning Goals

Across all the digital tools and resources analyzed, the number of tools decreased as a function of increasing level of learning goals. In other words, the higher the learning goal (eg, performing a strategic task through the process of guided discovery), the fewer tools or resources were available to support that goal. When analyzing within each of the learner groups individually, however, the academic-level learner group appeared to have more tools that supported the second-level learning goal of performing a procedural skill, followed by tasks focused on information acquisition and the performance of strategic skills. This aligns with the idea that learners at this level typically need to establish procedural skills (eg, learning how to administer a diagnostic assessment or how to score it) before they can be expected to apply these skills fluently and flexibly to multiple contexts or different patients. They benefit from highly structured, paced, and predefined frameworks within which practical skills can be explored [41]. The large number of tools and resources targeting the learning goal of information acquisition, although more receptive in nature, are useful for introductory learners with low content knowledge; these materials have been demonstrated to be effective in helping learners to link new knowledge with prior knowledge and thus may make new information more concrete, easier to integrate, and comprehend [54,55]. However, given that the ultimate goal of learning is to encourage greater guided discovery and train future professionals in more active, personal sense-making and critical thinking processes, it is discouraging to see that there are only a few digital tools and resources that target this learning goal. This learning goal is characterized by higher levels of learner interaction and lower levels of direct instruction; to become effective, independent health professionals, students need to become more independent self-guided learners [56].

Within the clinical-professional learner group, the trend of decreasing number of digital tools and resources as a function of increasing learning goal level was stark. There was a predominance of tools with the learning goal of receptive information acquisition, many of which consisted of continuing education opportunities. Although this is not particularly surprising, given the fact that clinical professionals are expected to have already attained a certain level of competency and often have limited time to attend such continuing education opportunities, it is nonetheless problematic that many tools only target these more *surface*-level learning goals; after all, performance of strategic skills through a guided discovery learning architecture is typically most beneficial for advanced learners (eg, beginning and even well experienced clinical professionals) who do not require a paced or scaffolded support [57]. Considering that clinical professionals are often expected to flexibly apply new information they learn from continuing educational opportunities without much prior practice directly to their complex caseloads, the question arises as to whether current digital continuing education opportunities truly foster effective lifelong learning [58,59]. As Scott et al [60] emphasized, measures must be implemented to aid retention and evaluate learning outcomes, not just to measure the satisfaction that professionals may have had with a virtual continuing education opportunity.

### Formats

Across all tools and resources, it appears that a large majority of tools are in visual format, followed by verbal and interactive formats. Although the large number of tools dedicated specifically to pictures or diagrams, text, and video is not particularly surprising given that these formats dominate the World Wide Web, it is notable that apps also contributed to a large proportion of all the tools. These primarily consisted of what we have termed *dynamic apps*, which involved a higher level of virtuality and interaction (eg, animations, virtuality, more integrated multimedia, and ability to manipulate components). Importantly, however, although an app is labeled as dynamic, this does not mean that its level of virtuality or interactivity is necessarily always the same among the different tools. An app involving 3D simulation and another app that displays animated procedures and only some interactive parts (eg, 3D manipulative or drawing tool) would still be considered dynamic interactive apps. It was beyond the scope of this initial investigation to study the full scope of virtuality and interaction of these tools, as these spectra are still being defined [48,61].

Within the academic-level–learner group, it was encouraging to see that there was a presence of more interactive formats, particularly dynamic apps and simulations; greater interaction is known to be associated with greater levels of learner engagement and thus motivation to promote learning and knowledge retention [62,63]. It is important to mention that many of these tools have not been evaluated for their efficacy. Thus, it would be useful to investigate whether these more interactive formats do indeed foster greater learner motivation, retention of information, or application to academic and clinical contexts (eg, does a simulation of a flexible endoscopic examination of swallowing necessarily translate to the appropriate motor skills to perform such a task in a clinical context?). Considering the current challenges in securing diverse clinical placements and externship experiences for students, it is critical to consider alternative methods for clinical training moving forward, including through digital means [64,65]. There is already evidence that simulation programs, for example, have some level of demonstrated efficacy for improving knowledge, skills, and confidence among health professional students [66-68]. Interactive formats can also serve as a useful platform from which one can begin training for professional skills such as interpersonal collaborative communication skills, which cannot be easily trained through only simple static visual or verbal formats [69].

Within the clinical-professional learner group, it appears that most digital learning resources and tools have relatively static verbal and visual formats at this time (predominated by picture or diagrams and text); there are additionally very few tools with interactive formats, a large portion of which consists of web-based courses or MOOCs, which makes sense given that many continuing education opportunities are currently also available virtually. However, the general dearth of interactive formats for clinical-professional learners points to an area of opportunity to spark greater engagement and more motivated lifelong learning.

Between the two groups, it was observed that in general, academic-level learners tended to overall have more *novel* formats than their clinical-professional learner counterparts (eg, in comparison traditional media such as text, audio, video, these are formats such as apps that have emerged since the 2000s) [70]. This was the case both in terms of within the interactive format subgroup (particularly for dynamic apps) and within the visual format subgroup (particularly for static apps and 3D models or manipulatives). However, considering that this study only investigated digital tools and resources for the purposes of learning, it could be that clinical professionals are rather using the apps for the purposes of clinical practice instead. There are studies that have discussed clinical apps (eg, diagnostic or therapeutic apps) for clinician and physician use [35,38,71]. In the areas of web-based courses or MOOCs, social networks, and portals, a greater proportion of tools and resources for clinical-professional use were found. As mentioned previously, the greater number of web-based courses or MOOCs could be explained by the fact that clinicians and physicians are required to complete continuing education credits, many of which are now web-based. The greater proportion of portals and field-specific social networking sites could be explained by the fact that many of the academic-level learners may still be learning about these field-specific resources in their graduate coursework and generationally, may be more drawn toward exchange on common social networking channels (eg, Facebook, Twitter, Instagram, and blogs). It will be interesting to see whether digital tool formats begin to consolidate between the 2 groups moving forward and to see what new digital formats begin to arise.

### Limitations

This study must be interpreted in light of its limitations. First, this initial investigation is not a fully comprehensive collection and analysis of all existing tools that are appropriate in the fields of SLP, phoniatrics, and otolaryngology. Given the specificity of the inclusion and exclusion criteria of this study, we intentionally did not investigate more collaborative digital learning spaces such as blogs or groups on common social media channels (eg, Facebook, Twitter, and Instagram), which are wide in scope and require their own critical investigation. Studies have shown that these seemingly *less academic* channels are an increasingly useful source of professional information and that even academic players (eg, institutions, regulating bodies, and peer-reviewed journals) are beginning to enter these spaces [72-75]. Therefore, it will be important to investigate these channels in future studies. Second, the tools and resources that have been investigated in this study reflect only one method for viewing or organizing digital tools and resources. Our findings are based on several theoretical models (eg, cognitive theory of multimedia learning by Mayer and the presentation forms by Arnold et al [41] as an organizational structure for digital format types) that we deemed appropriate and feasible on the basis of the nature of the tools and resources identified [40,47]. The division between different groups (eg, between academic-level learners vs clinical-professional learners or differentiation between static vs dynamic apps) was made to the best of our knowledge on the basis of a thorough review of previous literature, as many of these organizing structures are not currently well defined and are still developing. It should also be mentioned that the choice of search terms may have limited the range and type of tools that were found. However, these broader terms were chosen to maximize the search results. Considering the rapidly evolving nature of digitalization, this study presents just a snapshot of the digital tools and resources available at the time of the study. Therefore, not only is it difficult to exactly replicate study findings because of continuously changing search results, but the distinctions and definitions used in this study can also be interpreted as somewhat arbitrary in nature. Nevertheless, this study provides an emerging structure for better understanding the breadth and classifications of current digital tools and resources.

### Future Directions

Given this initial investigation into the organizing structures and availability of these tools and resources with open or good accessibility, it will be important as a next step to quantify their actual use. Investigating students’ and professionals’ attitudes toward such tools and resources is critical to understanding their use in practice or how they can be better incorporated into current curricula or learning opportunities. Perhaps most crucially, all digital resources and tools for teaching and learning need to undergo a process of rigorous peer review for quality assessment. In light of the digital revolution, tools such as the Mobile App Rating Scale have been developed to aid in the evaluation of digital applications, although gold standard measures or formal regulations supported by medical regulating bodies have yet to be developed or consistently implemented [76,77]. As standards are important for the processes of streamlining, compatibility, interchangeability, usability, and quality improvement and assurance, it is crucial that quality expectations become a greater area of focus, discussion, and productive problem solving in the future [78]. Although technical standards for e-learning apps are available from institutions such as the International Organization for Standardization, the Learning Technology Standards Committee of the Institute of Electrical and Electronics Engineers, or from the IMS Global Learning Consortium, Inc, it will be important—especially for the interdisciplinary fields of SLP, phoniatrics, and otolaryngology—to consider and begin to explicitly outline how these standards fit within current clinical-professional standards, roles, and responsibilities [79-81].

Furthermore, it will be critical to discuss the incorporation of digital skills into the clinical curricula, so that future professionals are better prepared for the changing medical landscape. The digital revolution has brought opportunities for innovation; however, innovation must be sustainable. As student and patient populations diversify and technologies progress, it is vital that health care professionals are robustly prepared to access, manipulate, critically assess, and improve digital tools and resources. To begin this process, this study presents an initial overview of the current digital landscape and organizing structures of the available tools and resources in fields related to communication disorders. However, there remains much work to be done.

## Appendix

Multimedia Appendix 1 Summary list of tools.

## Abbreviations

mHealth: mobile health
MOOC: massive online open course
SLP: speech-language pathology
