# Supplementary material for: Digital Learning in Speech-Language Pathology, Phoniatrics, and Otolaryngology: Interdisciplinary and Exploratory Analysis of Content, Organizing Structures, and Formats
Source: JMIR Med Educ. 2021 Jul 27;7(3):e27901. doi: 10.2196/27901 (PMC8367137; doi:10.2196/27901)
Supplement: Multimedia Appendix 1 [file mededu_v7i3e27901_app1.pdf]

Multimedia Appendix 1. Summary List of Tool

**Tool Name**	**Language**	**Learning Goal**	**Learner Group**	**Content**	**Format**	**Link**
A Practical Guide to Clinical Medicine: Head and Neck Exam	E	I	AC	D	T, P	A Practical Guide to Clinical Medicine: Head and Neck Exam
ABC of Ear, Nose and Throat 6e	E	I	AC	A, D, P, T	U	ABC of Ear, Nose and Throat 6e
AMBOSS	E, G	P2	AC	A, D, P, T	D	AMBOSS
Anatomy of the Larynx, Cartilage Structures	E	I	AC	A	V	Anatomy of the Larynx, Cartilage Structures
Anatomy of the Larynx, Online 3D	E	P1	AC	A	3D	Anatomy of the Larynx, Online 3D
Anatomy of the Inner Ear, Online 3D	E	P1	AC	A	3D	Anatomy of the Inner Ear, Online 3D
Atlas of Head & Neck Pathology: Ohio State University	E	I	AC	A, P	T, P	Atlas of Head & Neck Pathology: Ohio State University
AudCal HD	E	P1	AC	D	D	AudCal HD
Larynx ID	E	P1	AC	A	D	Larynx ID
Ear Disorders - Inner	E	P1	AC	P	D	Ear Disorders - Inner
Ear Disorders Outer & Middle	E	P1	AC	P	D	Ear Disorders Outer & Middle
Otitis Media Disorders	E	P1	AC	P	D	Otitis Media Disorders
Ear ID eBook	E	I	AC	A	U	Ear ID eBook
Ear ID	E	P1	AC	A	D	Ear ID
Hearing Anatomy	E	P1	AC	A	D	Hearing Anatomy
Inner Ear ID	E	P1	AC	A	D	Inner Ear ID
Middle Ear ID	E	P1	AC	A	D	Middle Ear ID
Laryngectomy	E	P1	AC	P	D	Laryngectomy
Vocal Pathology: Neurological	E	P1	AC	P	D	Vocal Pathology: Neurological
Vocal Pathology: Paresis/Paralysis	E	P1	AC	P	D	Vocal Pathology: Paresis/Paralysis
Vocal Pathology: Polyps	E	P1	AC	P	D	Vocal Pathology: Polyps
Vocal Pathology: Reflux	E	P1	AC	P	D	Vocal Pathology: Reflux
Larynx and Vocal Folds ID eBook	E	I	AC	A	U	Larynx and Vocal Folds ID eBook
Swallowing Oral Disorders	E	P1	AC	P	D	Swallowing Oral Disorders

Aspiration Disorders	E	P1	AC	P	D	Aspiration Disorders
Sinus ID	E	P1	AC	A	D	Sinus ID
Speech Articulation Apps, Affricates/Stops	E	P1	AC	A	D	Speech Articulation Apps, Affricates/Stops
Speech Articulation, Central/Diphthong	E	P1	AC	A	D	Speech Articulation, Central/Diphthong
Speech Articulation, Fricatives	E	P1	AC	A	D	Speech Articulation, Fricatives
Speech Articulation, Semivowels	E	P1	AC	A	D	Speech Articulation, Semivowels
Speech Articulation, Vowels Front Back	E	P1	AC	A	D	Speech Articulation, Vowels Front Back
Speech Articulation eBook	E	I	AC	A	U	Speech Articulation eBook
Swallowing ID	E	P1	AC	A	D	Swallowing ID
Vocal Folds ID	E	P1	AC	A	D	Vocal Folds ID
Buckingham Virtual Tympanum APP	E	I	AC	A, D, P	U	Buckingham Virtual Tympanum APP
CASUS	E, G	P2	AC	D, P, T	O	CASUS
Dartmouth Medicine: Human Anatomy Learning Modules	E	P1	AC	A	T, P, V	Dartmouth Medicine: Human Anatomy Learning Modules
eBooks Directory: Audiology and Speech Pathology	E	I	AC	A, D, P, T	T, P	eBooks Directory: Audiology and Speech Pathology
eDocTrainer	G	P2	AC	A, D, P, T	T, P	eDocTrainer
ENT e-Tutorial Clinical Guide	E	P1	AC	D	T, P, V	ENT e-Tutorial Clinical Guide
ENT Atlas and Guide	E	I	AC	P, T	U	ENT Atlas and Guide
ENT USA: Larynx Videos	E	P1	AC	P	T, P, V	ENT USA: Larynx Videos
ENT Ward Handbook	E	I	AC	A, D, P, T	T, P	ENT Ward Handbook
Focus Otolaryngology Understanding Disease	E	I	AC	D, P, T	U	Focus Otolaryngology Understanding Disease
Interactive Atlas of the Larynx	E	I	AC	A	T, P	Interactive Atlas of the Larynx
Interactive Ear: A Guide to Human Hearing	E	P1	AC	A	T, A	Interactive Ear: A Guide to Human Hearing
KenHub: "Head and Neck Anatomy" section	E	P1	AC	A	O	KenHub: "Head and Neck Anatomy" section
LearnENT	E	P1	AC	A, D, P, T	U	LearnENT

Lecturio Medical Education- Open lectures for ENT	E, G	P1	AC	A, D, P, T	T, P, V	Lecturio Medical Education- Open lectures for ENT
MIT Open Courseware: Speech Pathology	E	P1	AC	A, D, P, T	T, P	MIT Open Courseware: Speech Pathology
Mechanism of hearing educational VR 3D	E	P1	AC	A	D	Mechanism of hearing educational VR 3D
My Ear Anatomy	E	I	AC	A	U	My Ear Anatomy
Nat's Notes in Otolaryngology Head and Neck Surgery	E	I	AC	A, D, P, T	A	Nat's Notes in Otolaryngology Head and Neck Surgery
Oto Source: Comprehensive Otolaryngology Curriculum	E	I	AC	D ,P, T, PI	T	Oto Source: Comprehensive Otolaryngology Curriculum
See ENT	E	I	AC	A, D, P, T	U	See ENT
SFO-UK: ENT UK Podcast Series, Laryngology and Head and Neck Video Podcasts	E	I	AC	A, D, P, T, PI	A	SFO-UK: ENT UK Podcast Series, Laryngology and Head and Neck Video Podcasts
Simucase	E	P2	AC	A, D, P, T	S	Simucase
Sounds of Speech	E, G	P1	AC	A	D	Sounds of Speech
Three-D Ear	E	P1	AC	A	T, P, 3D	Three-D Ear
Upper Respiratory Virtual Lab (URVL)	E	P1	AC	A, D	S	Upper Respiratory Virtual Lab (URVL)
Voice Science Works	E	I	AC	A, T	T, P, V	Voice Science Works
WEVOSYS: RBH Learning and Practice	G	P2	AC	D, P	A, D	WEVOSYS: RBH Learning and Practice
WEVOSYS Medical: Spectro Real	G	P2	AC	D	D	WEVOSYS Medical: Spectro Real
AAO-HNSF Home Study Course	E	I	CP	PI	T, P, V	AAO-HNSF Home Study Course
AAO-HNSF FrequENTcy Podcasts	E	I	CP	D, P, T, PI	A	AAO-HNSF FrequENTcy Podcasts
Acute and Chronic Rhinosinusitis: A Comprehensive Review	E	I	CP	P	O	Acute and Chronic Rhinosinusitis: A Comprehensive Review
ASHA CEU Library and Learning Pass	E	P1	CP	D, T, P, PI	O	ASHA CEU Library and Learning Pass
ASHA Voices	E	I	CP	P, T, D, PI	A	ASHA Voices
American Head and Neck Society Surgical Videos	E	I	CP	T	V	American Head and Neck Society Surgical Videos

Aquifer (MedU): virtual cases (surgical ENT)	E	P1	CP	A, D, P	O	Aquifer (MedU): virtual cases (surgical ENT)
Broadcast Med: Otolaryngology	E	I	CP	P, T	V	Broadcast Med: Otolaryngology
COCLIA Curriculum from AAO-HNS	E	I	CP	D, P, T, PI	T, P	COCLIA Curriculum from AAO-HNS
Coursera: HPV-Associated Oral and Throat Cancers: What You Need to Know	E	I	CP	P, T	O	Coursera: HPV-Associated Oral and Throat Cancers: What You Need to Know
Coursera: Voice Disorders: What Patients and Professionals Need to Know	E	P1	CP	A, D, P, T	O	Coursera: Voice Disorders: What Patients and Professionals Need to Know
ENT Connect: Engage, Network, Transform	E	I	CP	O	X	ENT Connect: Engage, Network, Transform
ENT Expert Opinion Podcast	E	I	CP	D, P, T, PI	A	ENT Expert Opinion Podcast
ENT MasterClass	E	I	CP	PI	T, P, V	ENT MasterClass
HNO-Radiologie und Sonographie	G	I	CP	A, D	T, P	HNO-Radiologie und Sonographie
IALP Online Courses	E	I	CP	D, P, T	O	IALP Online Courses
IFOS/Developing World ENTOpen Access Atlas of Otolaryngology, Head & Neck Operative Surgery	E	I	CP	D, T	T, P	IFOS/Developing World ENTOpen Access Atlas of Otolaryngology, Head & Neck Operative Surgery
Live-International Otolaryngology Network (LION)	E	I	CP	P, T, PI, O	V	Live-International Otolaryngology Network (LION)
MERLOT: Communication Sciences & Disorders	E	I	CP	A, D, P, T	R	MERLOT: Communication Sciences & Disorders
Pediatric OTO Webinar Series (from AAOHN)	E	I	CP	D, P, T, PI	O	Pediatric OTO Webinar Series (from AAOHN)
SAGE Otolaryngology PODCAST	E	I	CP	A, D, P, T, PI	A	SAGE Otolaryngology PODCAST
SFORL with International Federation of Otorhino-Laryngological Societies (IFOS)	E, G	I	CP	A, D, P, T	T, P	SFORL with International Federation of Otorhino-Laryngological Societies (IFOS)
Sinus Videos: Sinus Surgery Videos Online	E	I	CP	A, D, T	V	Sinus Videos: Sinus Surgery Videos Online
Speechbite	E	I	CP	P, T, D, PI	R	Speechbite

Speechpathology.com	E	P1	CP	D, P, T, PI	O	Speechpathology.com
Springer Medizin CME	G	I	CP	A, D, P, T, PI	T, P	Springer Medizin CME
Thieme CME	G	I	CP	A, D, P, T, PI	T, P	Thieme CME
AirwayX	E	P2	AC, CP	A, D	S, D	AirwayX
AcademyQ, OtoLOGIC	E	P1	AC, CP	D, P, T, PI	D	AcademyQ, OtoLOGIC
aVOR	E	P1	AC, CP	A, D, P, T	D	aVOR
Bilinguistics	E, S	I	AC, CP	D, P, T	T, P	Bilinguistics
OTOQuest: Knowledge Assessment Tool	E	P2	AC, CP	A, D, P	T, P	OTOQuest: Knowledge Assessment Tool
ENT Surgery, David Luff	E	I	AC, CP	P	T, P, V	ENT Surgery, David Luff
E-lefENT: interactive learning platform	E	P1	AC, CP	D, P, T, PI	T, P, V	E-lefENT: interactive learning platform
ENT 3D Project	E	P1	AC, CP	A, D, T	T, P, V, 3D	ENT 3D Project
ENT Exam Video Series	E	I	AC, CP	D	P, V	ENT Exam Video Series
ENT Surgical Handbook	E	I	AC, CP	T	U	ENT Surgical Handbook
ENT USA: Larynx Videos	E	P1	AC, CP	A, D, P, T	A, T, P, V	ENT USA: Larynx Videos
ENTSHO	E	I	AC, CP	P, T	T, P	ENTSHO
Focus Otolaryngology Animated Pocket Dictionary	E	I	AC, CP	A, D, P, T	U	Focus Otolaryngology Animated Pocket Dictionary
Glossar HNO-Krankheiten	G	I	AC, CP	P, T	T, P	Glossar HNO-Krankheiten
Interactive CT Sinus Anatomy	E	I	AC, CP	A, D	T, P, V	Interactive CT Sinus Anatomy
KELDAMed: E-Learning Database	G	I	AC, CP	A, D, P, T, O	R	KELDAMed: E-Learning Database
King AbdulAziz University Hospital Voice Course	E	I	AC, CP	A, D, P, T	T, P	King AbdulAziz University Hospital Voice Course
Laryngopedia: Bastian Medical Media	E	I	AC, CP	P, T	A, T, P	Laryngopedia: Bastian Medical Media
Larynx and Voice Surgery Case Presentations	E	I	AC, CP	D, P, T	T, P	Larynx and Voice Surgery Case Presentations

LRSMed: Learning Resource Server Medizin	E, G	I	AC, CP	A, D, P, T, O	R	LRSMed: Learning Resource Server Medizin
Madde Synthesizer	E, G	P1	AC, CP	D	S	Madde Synthesizer
MedTube	E, G	I	AC, CP	D, P, T, PI	V	MedTube
National Center for Voice and Speech (NCVS)	E	I	AC, CP	A, D, P, T	T, P, V	National Center for Voice and Speech (NCVS)
Online Courses and Lectures (OCL) from the AAO-HNS	E	P1	AC, CP	D, P, T, PI	O	Online Courses and Lectures (OCL) from the AAO-HNS
Pulse QD	E	P1	AC, CP	O	X	Pulse QD
UTMB Online Otolaryngology	E	I	AC, CP	D, P, T, PI	T, P	UTMB Online Otolaryngology
Small Atlas of (Pediatric) Otoscopy	E	I	AC, CP	A	U	Small Atlas of (Pediatric) Otoscopy
SpeechTrainer: Visualisierung von Artikulationsbewegungen	E, G	P1	AC, CP	A, D	S, D	SpeechTrainer: Visualisierung von Artikulationsbewegungen
Stanford Otolaryngology: Head & Neck Surgery	E	I	AC, CP	D, P, T, PI	A	Stanford Otolaryngology: Head & Neck Surgery
Vocapedia	E	I	AC, CP	A, D, P, T	T, P	Vocapedia
Voice Analyst App	E	P1	AC, CP	P, D	D	Voice Analyst App
Vocal Tract Lab	E, G	P1	AC, CP	A, D	S	Vocal Tract Lab
Voice Doctor.net	E	I	AC, CP	A, D, P, T	T, P, V	Voice Doctor.net
Voice Foundation Educational Media	E	I	AC, CP	A, D, P	T, P, V	Voice Foundation Educational Media
Voce Vista	E	P1	AC, CP	D	S	Voce Vista

Tool Name: (name of tool)
Language: E = English; G = German; S= Spanish
Learning Goal: I = Inform, Receptive; P1 = Perform, Directive; P2 = Perform, Guided Discovery
Learner Group: AC = Academic-Level-Learners, CP = Clinical-Professional-Learners
Content: A = Anatomy & Physiology; D = Diagnostics, Evaluation; P = Pathology; T = Treatment; PI = Professional Issues; O = Other
Format: A = Audio; T = Text; P = Picture/Diagram; V = Video, 3D = 3D Model or manipulative; R = Portal, U = App, static; D = App Dynamic; S =Simulation; O = Online course or MOOC; X = Social Network
Link (These links were active at the time of analysis on October 28, 2020)
